# Pre- and post-diagnostic dairy intake in relation to recurrence and all-cause mortality in people with stage I-III colorectal cancer

**DOI:** 10.1007/s00394-023-03201-0

**Published:** 2023-07-02

**Authors:** Anne-Sophie van Lanen, Dieuwertje E. Kok, Evertine Wesselink, Renate M. Winkels, Henk K. van Halteren, Johannes H. W. de Wilt, Ellen Kampman, Fränzel J. B. van Duijnhoven

**Affiliations:** 1 Division of Human Nutrition and Health, Wageningen University & Research, Wageningen, The Netherlands; 2 Department of Internal Medicine, Admiraal de Ruyter Hospital, Goes, The Netherlands; 3 Department of Surgery, Radboud University Medical Center, Nijmegen, The Netherlands

**Keywords:** Low-fat dairy, High-fat dairy, Yoghurt, Colorectal cancer, Recurrence, All-cause mortality

## Abstract

**Purpose:**

Higher dairy consumption is associated with a lower risk of colorectal cancer (CRC), but no studies thus far have investigated its relation with recurrence in CRC. Few studies have investigated total dairy in relation to mortality in CRC, and yielded inconsistent results.

**Methods:**

In this prospective cohort study, people newly diagnosed with stage I-III CRC filled out a food frequency questionnaire at diagnosis (n = 1812) and six months after diagnosis (n = 1672). We examined associations between pre- and post-diagnostic intake of total dairy, low-fat dairy, high-fat dairy, milk, yoghurt, and cheese with recurrence and all-cause mortality using multivariable Cox proportional hazard models and restricted cubic splines (RCS).

**Results:**

A total of 176 recurrences and 301 deaths occurred during a median follow-up of 3.0 and 5.9 years, respectively. Before diagnosis, a higher low-fat dairy intake was associated with a lower risk of recurrence (HR_Q4vsQ1_: 0.42, 95% CI 0.26–0.67; P_RCS_: 0.008) and all-cause mortality (HR_Q4vsQ1_: 0.58, 95% CI 0.41–0.81; P_RCS_ < 0.001), whereas a higher high-fat dairy consumption tended to be associated with an increased all-cause mortality risk (HR_Q4vsQ1_: 1.41, 95% CI 0.98–2.01; P_RCS_: 0.030). After diagnosis, only the associations between low- and high-fat dairy in relation to all-cause mortality remained.

**Conclusions:**

This study demonstrated that higher pre- and post-diagnostic intakes of low-fat dairy were associated with a reduced all-cause mortality risk in people with stage I-III CRC, whereas higher intakes of high-fat dairy were associated with an increased all-cause mortality risk. Also, a higher pre-diagnostic low-fat dairy intake was associated with a reduced risk of recurrence.

**Trial registration:**

Clinical Trials.gov identifier: NCT03191110.

**Supplementary Information:**

The online version contains supplementary material available at 10.1007/s00394-023-03201-0.

## Introduction

Evidence from observational studies suggests that dairy consumption decreases the risk of colorectal cancer (CRC) [1, 2]. Contrasting the large body of evidence and the proposed biological mechanisms [3, 4] available on dairy consumption and CRC risk, a scarcity of studies has been published on the role of dairy intake in CRC prognosis, such as cancer recurrence and all-cause mortality.

For total dairy, two large prospective cohort studies and one pooled analysis of prospective cohort studies observed no association between pre-diagnostic [5, 6] or post-diagnostic [6, 7] dairy intake and CRC-specific or all-cause mortality. However, when stratifying for fat content, one pooled analysis including 1753 individuals with CRC showed that a higher post-diagnostic intake of low-fat dairy products was associated with a reduced risk of all-cause mortality, whereas a higher post-diagnostic intake of high-fat dairy products was associated with an increased risk of all-cause mortality [7]. The authors speculated that potentially beneficial dairy components may be counteracted by fat or fat-related components of dairy [7], which may explain previously observed null associations for total dairy intake with all-cause mortality [5, 6]. We therefore hypothesise that low-fat dairy intake is associated with a reduced risk of all-cause mortality, and that high-fat dairy intake is associated with an increased risk of all-cause mortality.

Inherent to their unique nutritional compositions and in line with existing literature on CRC risk, different dairy products, such as milk, yoghurt, and cheese, are proposed to have a differential influence on CRC prognosis [1, 2, 8–10]. So far, not enough studies have been performed to draw firm conclusions on the association between specific dairy product intake and mortality in CRC. To the best of our knowledge, it is yet unknown whether dairy intake before or after diagnosis is associated with cancer recurrence in people with CRC.

Therefore, we investigated the relation between pre- and post-diagnostic intake of dairy (total, low-fat, high-fat) and specific dairy products (milk, yoghurt, cheese) with cancer recurrence and all-cause mortality in a prospective cohort of newly-diagnosed males and females with stage I-III CRC.

## Materials and methods

### Study cohort

**Fig. 1:**
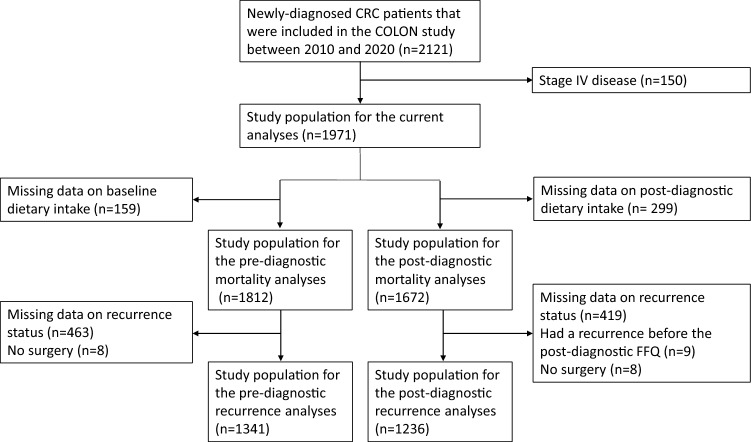
Flowchart representing participant selection for the data analyses

For these analyses we used data from adults with newly diagnosed CRC who participated in the ‘COlorectal cancer: Longitudinal, Observational study on Nutritional and lifestyle factors that influence colorectal tumour cancer recurrence, survival and quality of life’ (COLON) study (NCT03191110, ClinicalTrials.gov). Participants were recruited from 11 hospitals in the Netherlands between August 2010 and February 2020, and had no hereditary CRC syndrome, a history of CRC or inflammatory bowel disease [11]. Details of this study have been described previously [11]. For the current analyses, we excluded some participants (Fig. 1), among which people diagnosed with stage IV disease (n = 150), as we expect the potential role of lifestyle to be very limited in the poor prognosis of these persons. The final analytical sample sizes were as follows: 1812 (all-cause mortality) and 1341 (recurrence) for pre-diagnostic models, and 1672 (all-cause mortality) and 1236 (recurrence) for post-diagnostic models (Fig. 1). Three participants were lost to follow-up due to moving abroad. The COLON study was approved by a medical ethics committee (region Arnhem-Nijmegen, 2009–349), and all study participants provided written informed consent.

### Dietary assessment

Upon diagnosis, participants filled out a semi-quantitative food frequency questionnaire (FFQ) of 204 items [12, 13], reflecting dietary intake in the month prior to diagnosis. The exact date of diagnosis was not uniformly available, but in practice participants were included in the study by the health care practitioner only a few days after the diagnosis. The median time between this date of inclusion and completion of the first FFQ was 5 days (IQR: 3–10). At six months after diagnosis participants also completed the FFQ, reflecting dietary intake in the month prior to filling out the FFQ. Dairy product intakes and energy intakes per day were calculated, based on frequency of intake and portion sizes and using the online Dutch Food Composition Table [14], and categorized into total, low-fat and high-fat dairy. Total dairy comprised of all dairy products included in low- and high-fat dairy. Low-fat dairy included low-fat or skimmed versions of milk, yoghurt, custard, and soft curd cheese, and buttermilk. High-fat dairy included whole and condensed milk, full-fat versions of yoghurt, custard and soft curd cheese, all other cheeses, ice cream, whipped cream, and butter. Total milk included all plain and sweetened milk, regardless of fat content. Ready-made breakfast drinks were not included, because not all ready-made breakfast drinks on the Dutch market contain dairy. Total yoghurt included all plain and sweetened yoghurt, yoghurt drinks and soft curd cheese, regardless of fat content. Total cheese included all hard and soft cheese, except for soft curd cheese. Intake of dairy types and products was adjusted for total energy intake using the energy residual method [15]. Further assessment of individual food items, such as skimmed milk, low-fat milk and high-fat milk, was not possible due to low median intakes and a low variation in intake. To improve interpretability, the predicted dairy intake at the median total energy intake was added to individual residuals.

### Study outcomes

Recurrence was defined as a local or regional cancer recurrence or a distant metastasis diagnosed after initial tumour resection. Recurrence data were requested from the Netherlands Cancer Registry via the Netherlands Comprehensive Cancer Organisation (IKNL), most recently updated in February 2018. All-cause mortality data were obtained from the municipal registrations (BRP), most recently updated in September 2021.

For recurrence, follow-up time was calculated starting from date of surgery until date of recurrence, until date of death, until the date recurrence status was updated, or until lost to follow-up, whichever came first. Follow-up time for mortality was defined as starting from date of surgery until date of death, until the last date mortality status was updated, or until lost to follow-up, whichever came first. If date of surgery was unavailable (n = 71), date of filling out the questionnaires was used. For post-diagnostic analyses, follow-up time was calculated starting from date of filling out the post-diagnostic questionnaires.

### Assessment of covariates

At diagnosis and six months after diagnosis, participants filled out a general questionnaire on demographics, anthropometrics, and lifestyle habits, including questions about age (continuous), sex (male/female), education (low, medium, high), body weight (kg), height (cm), smoking status (current, former, never), and calcium and vitamin D supplement use (including multivitamins, yes/no). Physical activity was assessed using the Short QUestionnaire to ASsess Health-enhancing physical activity (SQUASH) [16]. Moderate-to-vigorous physical activity (hours/week) included all activities with a metabolic equivalent value ≥ 3 [17]. Clinical data, such as disease stage (I-III), tumour location (proximal colon (caecum to the transverse colon), distal colon (splenic flexure to the sigmoid colon), rectum (rectosigmoid junction and rectum)), American Society of Anesthesiologists (ASA) classification (I-IV) and type of treatment (only surgery, surgery and chemotherapy, surgery and radiotherapy, surgery and chemotherapy and radiotherapy, no or unknown surgery, unknown treatment), were collected via the Dutch ColoRectal Audit [18].

To assess possible confounding by other dietary factors previously associated with CRC risk [2], we also calculated total dietary intake of wholegrains, red meat, processed meat, dietary fiber, and alcohol, as defined in [2].

### Statistical analysis

Population characteristics are presented as medians [interquartile range (IQR)] or numbers (percentage). Sex-specific quartiles of pre- and post-diagnostic dairy intake were calculated.

Cox proportional hazards regression analyses were used to calculate Hazard Ratios (HR) and 95% Confidence Intervals (95%CI) for the associations between pre- and post-diagnostic dairy intake, and recurrence and all-cause mortality. No violation of the proportionality assumption for the Cox models was observed when visually inspecting log–log curves. For categorical analyses, the lowest quartile was used as the reference category. P_trend_ values were computed over quartiles of dairy intake using the medians of the corresponding quartiles. For continuous analyses increments were 100 g/day for total dairy, low-fat dairy, high-fat dairy, milk, and yoghurt, and 30 g/day for cheese. We first created a crude model with the exposure and outcome variables, that was adjusted for age and sex. Then, potential confounders were added to the model one by one, and were included when they changed the HR with > 10%. Based on literature, the following covariates were evaluated: disease stage, primary tumour location, BMI, education level, smoking status, moderate-to-vigorous physical activity, use of calcium supplements, use of vitamin D supplements, and total dietary intake of wholegrains, dietary fiber, red meat, processed meat and alcohol. Additionally, for the post-diagnostic dairy intake analyses, having a stoma, type of treatment, and BMI, smoking status, supplement use and dietary intake at 6 months post-diagnosis were evaluated for confounding. Finally, the adjusted model included age, sex, stage of disease and total energy intake (as part of the energy adjustment method [15]).

We also evaluated restricted cubic splines (RCS) to study linearity of the associations between dairy exposures and CRC prognosis, using the adjusted model. Knots were placed at the 5th, 50th, and 95th percentiles, and the graphs were truncated at the 1st and 99th percentile. The median intake of the first quartile of each exposure was used as the reference.

For linear associations, HRs are displayed continuously as well as in quartiles with p for trend, while for non-linear associations, only quartiles of dairy intake are displayed. All associations are visualized in plots.

Statistical analyses were performed using R Statistical Software (version 4.0.5). P-values below 0.05 and 95% confidence intervals not containing 1 were considered statistically significant.

## Results

### Population characteristics

Participants were on average 66 years (IQR: 61–72) at CRC diagnosis, and 37% was female (Table 1). Median BMI was 26.2 kg/m^2^ (IQR: 24.0–28.7) and 10% of participants were current smokers. Furthermore, 66% had a tumour located in the colon, and stage III (43%) was more common than stage I (26%) or stage II (27%) (Table 1).

**Table 1:** Population characteristics of people with colorectal cancer stratified by sex-specific quartiles of pre-diagnostic energy-adjusted intake of total dairy, low-fat dairy and high-fat dairy

Characteristics	Total population (n = 1812)	Quartile of energy-adjusted dairy intake					
		Total dairy		Low-fat dairy		High-fat dairy	
		Q1 (n = 454)	Q4 (n = 454)	Q1 (n = 454)	Q4 (n = 454)	Q1 (n = 454)	Q4 (n = 454)
Age, years, median [IQR]	66 [61–72]	64 [59–70]	68 [63–74]	66 [61–71]	67 [61–74]	64 [59–69]	67 [63–73]
Female sex, No. (%)	666 (36.8)	167 (36.8)	167 (36.8)	167 (36.8)	167 (36.8)	167 (36.8)	167 (36.8)
BMI, kg/m^2^, median [IQR]	26.2 [24.0–28.7]	26.3 [24.1–28.8]	26.2 [24.0–28.7]	25.8 [23.7–28.3]	26.2 [24.1–28.7]	25.9 [23.9–28.4]	25.4 [23.2–28.1]
Waist-hip-ratio, median [IQR]^a^	0.95 [0.90–1.00]	0.95 [0.90–1.00]	0.95 [0.90–1.00]	0.95 [0.90–1.00]	0.95 [0.90–1.00]	0.95 [0.89–1.01]	0.94 [0.90–1.00]
Level of education, No. (%)^b^							
Low	763 (42.1)	177 (39.0)	203 (44.7)	185 (40.7)	182 (40.1)	166 (36.6)	221 (48.7)
Medium	454 (25.1)	120 (26.4)	112 (24.7)	118 (26.0)	111 (24.4)	124 (27.3)	105 (23.1)
High	591 (32.6)	156 (34.4)	136 (30.0)	150 (33.0)	159 (35.0)	163 (35.9)	125 (27.5)
Unknown	4 (0.2)	1 (0.2)	3 (0.7)	1 (0.2)	2 (0.4)	1 (0.2)	3 (0.7)
Smoking status, No. (%)							
Current	184 (10.2)	65 (14.3)	40 (8.8)	69 (15.2)	33 (7.3)	41 (9.0)	57 (12.6)
Former	1057 (58.3)	263 (57.9)	265 (58.4)	253 (55.7)	261 (57.5)	269 (59.3)	254 (55.9)
Never	566 (31.2)	125 (27.5)	147 (32.4)	131 (28.9)	158 (34.8)	143 (31.5)	141 (31.1)
Unknown	5 (0.3)	1 (0.2)	2 (0.4)	1 (0.2)	2 (0.4)	1 (0.2)	2 (0.4)
Moderate-to-vigorous physical activity, hours/week, median [IQR] ^c^	11 [6–20]	11 [6–20]	12 [5–21]	11 [6–20]	12 [6–19]	12 [6–20]	11 [5–19]
Calcium supplement use, No. (%)^d^	313 (17.3)	92 (20.3)	67 (14.8)	83 (18.3)	82 (18.1)	79 (17.4)	64 (14.1)
Vitamin D supplement use, No. (%)	482 (26.6)	119 (26.2)	122 (26.9)	114 (25.1)	139 (30.6)	132 (29.1)	107 (23.6)
Location of the tumor, No. (%)^e^							
Proximal colon	549 (30.3)	133 (29.3)	149 (32.8)	132 (29.1)	133 (29.3)	127 (28.0)	149 (32.8)
Distal colon	650 (35.9)	164 (36.1)	164 (36.1)	170 (37.4)	179 (39.4)	173 (38.1)	142 (31.3)
Rectum	552 (30.5)	143 (31.5)	130 (28.6)	136 (30.0)	128 (28.2)	133 (29.3)	152 (33.5)
Unknown	61 (3.4)	14 (3.1)	11 (2.4)	16 (3.5)	14 (3.1)	21 (4.6)	11 (2.4)
Disease stage, No. (%)							
I	471 (26.0)	128 (28.2)	132 (29.1)	123 (27.1)	121 (26.7)	116 (25.6)	119 (26.2)
II	490 (27.0)	104 (22.9)	122 (26.9)	108 (23.8)	128 (28.2)	125 (27.5)	133 (29.3)
III	770 (42.5)	200 (44.1)	183 (40.3)	203 (44.7)	185 (40.7)	187 (41.2)	190 (41.9)
Unknown	81 (4.5)	22 (4.8)	17 (3.7)	20 (4.4)	20 (4.4)	26 (5.7)	12 (2.6)
Type of treatment, No. (%)^f^							
Surgery only	962 (53.1)	241 (53.1)	256 (56.4)	236 (52.0)	248 (54.6)	237 (52.2)	242 (53.3)
Surgery + chemotherapy	405 (22.4)	104 (22.9)	87 (19.2)	111 (24.4)	91 (20.0)	108 (23.8)	93 (20.5)
Surgery + radiotherapy	189 (10.4)	40 (8.8)	55 (12.1)	38 (8.4)	50 (11.0)	38 (8.4)	59 (13.0)
Surgery + chemotherapy + radiotherapy	182 (10.0)	52 (11.5)	42 (9.3)	50 (11.0)	47 (10.4)	49 (10.8)	47 (10.4)
No or unknown surgery	70 (3.9)	17 (3.7)	12 (2.6)	19 (4.2)	17 (3.7)	22 (4.8)	13 (2.9)
Unknown treatment	4 (0.2)	0 (0.0)	2 (0.4)	0 (0.0)	1 (0.2)	0 (0.0)	0 (0.0)
ASA classification, No. (%)							
I	502 (27.7)	135 (29.7)	120 (26.4)	138 (30.4)	106 (23.3)	133 (29.3)	135 (29.7)
II	959 (52.9)	226 (49.8)	249 (54.8)	222 (48.9)	262 (57.7)	242 (53.3)	231 (50.9)
III	204 (11.3)	48 (10.6)	58 (12.8)	52 (11.5)	51 (11.2)	38 (8.4)	58 (12.8)
IV	4 (0.2)	1 (0.2)	1 (0.2)	1 (0.2)	1 (0.2)	1 (0.2)	2 (0.4)
Unknown	143 (7.9)	44 (9.7)	26 (5.7)	41 (9.0)	34 (7.5)	40 (8.8)	28 (6.2)
Dietary intake, median [IQR]							
Total energy intake, kcal/day	1810 [1506–2172]	1899 [1520–2237]	1854 [1546–2261]	1884 [1517–2209]	1851 [1568–2201]	2028 [1703–2356]	1837 [1517–2189]
Energy-adjusted total dairy, g/day	270 [177–383]	114 [72–146]	481 [423–584]	131 [76–200]	454 [388–560]	232 [117–352]	327 [231–450]
Energy-adjusted low-fat dairy, g/day^g^	165 [70–281]	44 [9–83]	369 [283–455]	20 [-5–45]	372 [315–455]	212 [96–321]	110 [33–216]
Energy-adjusted high-fat dairy, g/day^h^	77 [46–126]	59 [28–91]	96 [55–186]	96 [56–166]	64 [38–99]	24 [5–37]	184 [150–246]
Energy-adjusted milk, g/day^i^	50.4 [14.7–141.8]	16.0 [-8.0–37.3]	188.3 [56.1–292.3]	19.1 [-3.7–41.9]	167.2 [43.1–281.6]	34.4 [-2.3–132.6]	66.6 [20.9–170.6]
Energy-adjusted yoghurt, g/day^j^	72.2 [19.5–139.3]	20.6 [3.1–61.1]	140.8 [55.0–250.0]	20.2 [2.7–66.6]	131.1 [51.2–225.3]	60.9 [8.5–142.6]	92.1 [35.6–151.7]
Energy-adjusted cheese, g/day^k^	27.7 [15.8–43.0]	22.3 [11.8–40.3]	29.6 [17.8–44.4]	26.4 [14.3–45.7]	26.2 [14.9–40.0]	17.8 [8.8–27.5]	32.3 [18.3–51.7]
Dietary fiber, g/day	19.7 [15.9–24.2]	19.7 [15.6–23.9]	19.9 [16.0–24.6]	19.3 [15.3–23.4]	20.4 [16.7–24.9]	22.5 [19.3–26.8]	18.9 [14.8–24.0]
Wholegrains, g/day	111.1 [75.4–150.8]	110.3 [74.0–151.3]	107.9 [75.6–149.5]	107.4 [68.4–150.9]	113.9 [78.5–155.6]	129.4 [85.8–172.1]	104.6 [71.3–145.2]
Red meat, g/day	35.7 [20.5–49.8]	36.8 [21.4–53.6]	34.8 [20.0–48.2]	35.9 [19.6–51.4]	35.7 [20.4–48.5]	37.7 [24.7–52.0]	34.5 [20.9–48.5]
Processed meat, g/day	28.1 [13.3–44.6]	33.6 [16.3–51.7]	25.1 [12.8–41.8]	30.5 [14.9–45.6]	26.3 [13.7–45.9]	35.0 [16.4–53.4]	25.0 [11.8–40.3]
Alcohol, g/day	8.1 [1.0–20.4]	13.0 [1.9–26.4]	4.6 [0.5–14.2]	9.6 [0.8–23.3]	5.6 [0.9–16.6]	12.7 [2.4–26.7]	4.4 [0.3–14.2]
Dietary calcium, mg/day	855 [650–1087]	638 [495–833]	1145 [938–1364]	689 [520–916]	1087 [898–1310]	830 [625–1055]	951 [770–1207]

*ASA* American Society of Anesthesiologists, *IQR* interquartile range

^a^Data were missing for 4 participants

^b^Low education was defined as primary school and lower general secondary education; medium as lower vocational training and higher general secondary education; high as higher vocational training and university

^c^Moderate-to-vigorous physical activity included all activities with a metabolic equivalent value ≥ 3 [17]. Data were missing for 8 participants

^d^Data were missing for 1 participant

^e^Proximal colon includes the caecum, appendix, ascending colon, hepatic flexure, and transverse colon. Distal colon includes the splenic flexure, descending colon, and sigmoid colon. Rectum includes the rectosigmoid junction and rectum

^f^Treatment includes neoadjuvant and adjuvant treatment

^g^Low-fat dairy included buttermilk and low-fat, reduced-fat or skimmed versions of milk, yoghurt, custard, and soft curd cheese

^h^High-fat dairy included whole and condensed milk, full-fat versions of yoghurt, custard and soft curd cheese, all other cheeses, ice cream, whipped cream, and butter

^i^Total milk included all plain and sweetened milk, regardless of fat content

^j^Total yoghurt included all plain and sweetened yoghurt, yoghurt drinks and soft curd cheese, regardless of fat content

^k^Total cheese included all hard cheese and soft cheese, except for soft curd cheese

Participants in the highest quartile of total pre-diagnostic dairy intake were older, less often current smokers, and more often had a lower level of education, compared to participants in the lowest quartile of total dairy products. Also, they consumed less processed meat and alcohol, consumed more dietary calcium, and less often used calcium supplements. Participants in the highest quartile of low-fat dairy intake were less often current smokers, consumed more dietary calcium and less alcohol, more often used vitamin D supplements, and less often had an ASA score of I, compared to participants in the lowest quartile. Participants in the highest quartile of high-fat dairy products were older and more often had a lower level of education, consumed more dietary calcium, and less processed meat and alcohol and had a lower total energy intake than participants in the lowest quartile (Table 1). Median pre-diagnostic energy-adjusted intakes of total, low-fat and high-fat dairy were 270 (IQR: 177–383), 165 (IQR: 70–281), and 77 (IQR: 46–126) g/day, respectively (Table 1). Median post-diagnostic energy-adjusted intakes of total, low-fat and high-fat dairy were 282 (IQR: 184–392), 161 (IQR: 70–286), and 81 (IQR: 48–136) g/day, respectively (data not shown). Pearson correlation coefficients between pre- and post-diagnostic intakes ranged between 0.41 and 0.61 (data not shown).

For pre-diagnostic analyses, we observed 176 recurrences during a median follow-up time of 3.0 years (IQR: 2.0–4.2), and 301 participants died during a median follow-up of 5.9 years (IQR: 3.9–7.2). For post-diagnostic analyses, we observed 153 recurrences that occurred during a median follow-up time of 2.6 years (IQR: 1.7–3.8), and 245 participants died during a median follow-up of 5.5 years (IQR: 3.8–6.8).

### Pre-diagnostic dairy intake in relation to recurrence and all-cause mortality

**Fig. 2:**
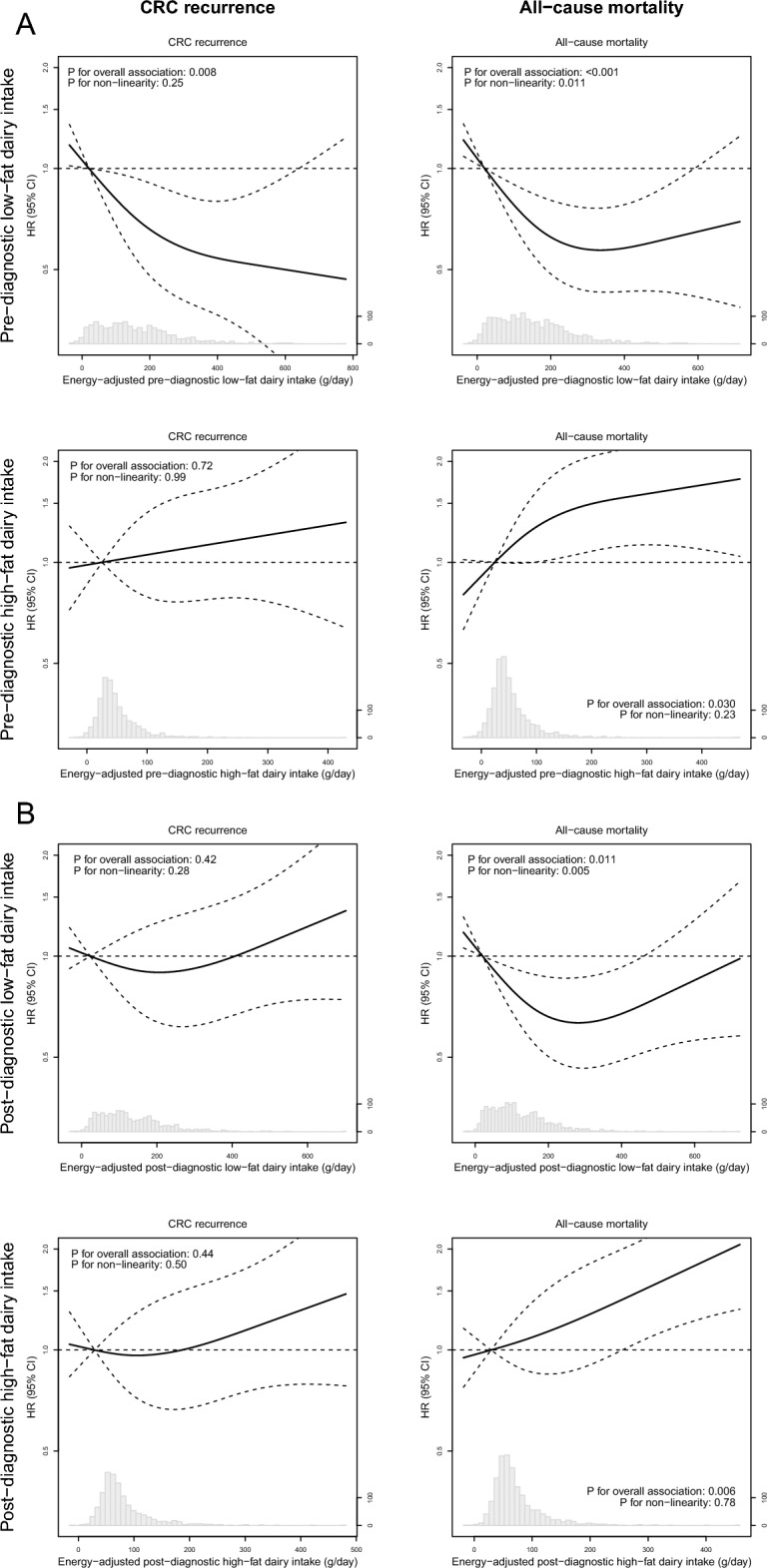
Associations between pre-diagnostic (**A**) and post-diagnostic (**B**) intake of low- and high-fat dairy and colorectal cancer prognosis. Solid lines are restricted cubic splines, and dashed lines are 95% confidence intervals. The reference values were set at the median of the first quartile of dairy intake. Knots were located at the 5th, 50th and 95th percentile, and the graphs were truncated at the 1st and 99th percentile. Analyses are adjusted for age, sex, disease stage and energy intake

As shown in Table 2 and Supplementary Fig. 1, when comparing the highest to the lowest quartile, a higher pre-diagnostic intake of total dairy was associated with a lower risk of recurrence (HR: 0.51, 95% CI 0.33–0.80; P_RCS_: 0.06), and tended to be associated with all-cause mortality (HR_Q4 vs Q1_: 0.72, 95% CI 0.52–1.01; P_RCS_: 0.18). A higher intake of low-fat dairy before diagnosis was associated with a lower risk of recurrence (HR: 0.42, 95%CI 0.26–0.67, P_RCS_: 0.008; Table 2 and Fig. 2A) and all-cause mortality (HR_Q4 vs Q1_: 0.58, 95% CI 0.41–0.81; P_RCS_ < 0.001). RCS revealed a significant non-linear association between pre-diagnostic low-fat dairy intake and all-cause mortality, with intakes higher than the median of the first quartile (i.e. 21 g/day) associated with a reduced risk of mortality (Fig. 2A). Contrastingly, a higher intake of high-fat dairy before diagnosis tended to be associated with an increased risk of all-cause mortality (HR_Q4 vs Q1_: 1.41, 95% CI 0.98–2.01; P_RCS_: 0.030). Pre-diagnostic high-fat dairy intake was not associated with recurrence. A higher yoghurt intake before diagnosis seemed associated with a lower risk of all-cause mortality (HR_Q4 vs Q1_: 0.70, 95% CI 0.50–0.99; P_RCS_: 0.09), but not with recurrence. No associations were observed between total milk or cheese intake before diagnosis and recurrence or all-cause mortality.

**Table 2:** Associations of pre-diagnostic energy-adjusted intake of total dairy, low-fat dairy, high-fat dairy, and dairy products with recurrence (n = 1315) and all-cause mortality (n = 1731) in sex-specific quartiles of people with stage I-III colorectal cancer

Dietary variable	Recurrence^a^				All-cause mortality			
	Median energy-adjusted intake [IQR]^b^	n	No. of recurrences/person-years	Multivariable HR (95% CI)^c^	Median energy-adjusted intake [IQR]^b^	n	No. of deaths/person-years	Multivariable HR (95% CI)^c^
Total dairy								
Q1	109.8 [69.7, 142.4]	331	55/1033	1.00	114.3 [73.0, 147.3]	432	74/2534	1.00
Q2	220.3 [184.3, 253.9]	330	46/1063	0.80 (0.54–1.19)	224.5 [190.0, 247.3]	432	78/2504	0.98 (0.71–1.35)
Q3	323.2 [278.1, 351.3]	326	42/1049	0.72 (0.48–1.08)	322.5 [279.1, 348.9]	430	64/2485	0.77 (0.55–1.09)
Q4	489.6 [426.1, 591.02]	328	30/1106	0.51 (0.33–0.80)	481.0 [423.7, 581.2]	437	66/2582	0.72 (0.52–1.01)
P_trend_	NA	NA	NA	0.003	NA	NA	NA	0.029
Continuous (per 100 g/day)	267.9 [172.1, 385.4]	1315	173/4251	0.89 (0.81–0.99)	269.6 [177.6, 384.6]	1731	282/10105	0.96 (0.89–1.03)
Low-fat dairy^d^								
Q1	20.5 [− 4.8, 42.3]	333	56/1076	1.00	20.5 [− 3.5, 44.6]	434	87/2511	1.00
Q2	120.1 [94.8, 142.0]	329	46/1034	0.83 (0.56–1.23)	119.7 [94.9, 143.7]	438	74/2503	0.84 (0.62–1.15)
Q3	223.0 [184.0, 254.9]	325	47/1035	0.84 (0.57–1.24)	219.6 [185.9, 253.4]	425	63/2474	0.69 (0.50–0.96)
Q4	374.3 [317.8, 465.4]	328	24/1108	0.42 (0.26–0.67)	371.9 [315.4, 452.4]	434	58/2618	0.58 (0.41–0.81)
P_trend_	NA	NA	NA	< .001	NA	NA	NA	< 0.001
Continuous (per 100 g/day)	163.3 [68.2, 281.4]	1315	173/4251	0.86 (0.77–0.96)	164.8 [69.7, 281.2]	1731	282/10105	*
High-fat dairy^e^								
Q1	25.4 [6.3, 36.9]	328	45/1044	1.00	24.5 [5.5, 36.9]	428	49/2553	1.00
Q2	60.7 [50.9, 68.7]	329	38/1065	0.80 (0.51–1.24)	61.3 [51.5, 69.0]	430	67/2525	1.13 (0.78–1.66)
Q3	96.7 [85.9, 109.2]	325	43/1068	0.93 (0.60–1.44)	96.8 [86.2, 109.2]	431	80/2483	1.33 (0.92–1.93)
Q4	180.3 [149.6, 229.8]	333	47/1075	0.97 (0.64–1.47)	182.9 [149.8, 244.3]	442	86/2545	1.41 (0.98–2.01)
P_trend_	NA	NA	NA	0.83	NA	NA	NA	0.06
Continuous (per 100 g/day)	76.0 [45.6, 128.5]	1315	173/4251	1.07 (0.91–1.26)	77.7 [46.5, 127.9]	1731	282/10105	1.15 (1.03–1.28)
Total milk								
Q1	− 4.2 [-19.1, 7.1]	331	43/1055	1.00	− 4.9 [-19.4, 6.7]	424	60/2508	1.00
Q2	31.8 [21.2, 41.4]	328	51/984	1.06 (0.69–1.64)	30.9 [21.1, 40.5]	432	75/2399	1.07 (0.75–1.55)
Q3	93.8 [69.6, 120.4]	330	40/1100	0.89 (0.57–1.40)	89.6 [67.4, 116.5]	439	74/2558	1.09 (0.76–1.55)
Q4	231.5 [175.1, 304.3]	326	39/1112	0.81 (0.52–1.27)	227.0 [171.1, 297.5]	436	73/2641	0.91 (0.64–1.29)
P_trend_	NA	NA	NA	0.23	NA	NA	NA	0.39
Continuous (per 100 g/day)	52.2 [15.3, 145.8]	1315	173/4251	0.90 (0.78–1.04)	51.2 [15.5, 142.5]	1731	282/10105	1.02 (0.92–1.12)
Total yoghurt								
Q1	2.4 [− 7.1, 9.9]	325	43/1088	1.00	2.4 [− 6.1, 11.3]	427	78/2531	1.00
Q2	45.8 [22.2, 62.0]	328	48/1040	1.14 (0.75–1.72)	45.3 [22.5, 68.0]	429	80/2449	0.91 (0.66–1.25)
Q3	102.3 [76.1, 122.0]	333	49/1074	1.09 (0.72–1.64)	102.3 [76.4, 124.7]	438	68/2588	0.74 (0.53–1.02)
Q4	205.0 [147.1, 277.2]	329	33/1049	0.78 (0.50–1.24)	204.6 [147.5, 273.6]	437	56/2537	0.70 (0.50–0.99)
P_trend_	NA	NA	NA	0.21	NA	NA	NA	0.029
Continuous (per 100 g/day)	70.8 [18.5, 138.4]	1315	173/4251	0.96 (0.83–1.13)	72.8 [19.9, 140.1]	1731	282/10105	0.86 (0.75–0.99)
Total cheese								
Q1	9.0 [2.5, 12.6]	329	47/1065	1.00	8.6 [2.4, 12.4]	429	63/2518	1.00
Q2	21.6 [19.4, 25.1]	325	35/1048	0.76 (0.49–1.18)	21.5 [19.0, 24.8]	429	64/2523	0.92 (0.65–1.32)
Q3	34.8 [31.1, 38.1]	331	47/1007	0.97 (0.64–1.46)	34.5 [31.1, 37.4]	436	78/2449	1.08 (0.77–1.51)
Q4	57.2 [49.1, 73.3]	330	44/1131	0.91 (0.60–1.38)	57.6 [49.1, 74.0]	437	77/2615	1.04 (0.75–1.46)
P_trend_	NA	NA	NA	0.95	NA	NA	NA	0.61
Continuous (per 30 g/day)	28.0 [16.1, 43.7]	1315	173/4251	1.03 (0.85–1.24)	27.8 [15.9, 43.2]	1731	282/10105	1.03 (0.89–1.19)

*CI* confidence interval, *HR* hazard ratio, *IQR* inter-quartile range. Cut-off points for female quartiles were as follows: 220.8, 308.1 and 441.3 g/day for total dairy; 101.4, 201.4 and 313.5 g/day for low-fat dairy; 58.0, 84.0 and 129.1 g/day for high-fat dairy; 23.6, 60.0 and 149.6 g/day for milk; 40.7, 104.6 and 164.6 g/day for yoghurt; 17.9, 29.8 and 44.8 g/day for cheese. Cut-off points for male quartiles were as follows: 152.1, 244.1 and 358.0 g/day for total dairy; 56.6, 145.7 and 264.0 g/day for low-fat dairy; 38.0, 72.5 and 123.1 g/day for high-fat dairy; 9.4, 46.0 and 137.2 g/day for milk; 9.7, 54.7 and 120.5 g/day for yoghurt; 14.3, 26.2 and 41.9 g/day for cheese

^*^This association was observed to be non-linear in restricted cubic splines. See Fig. 2 for the continuous analysis

^a^Recurrence is defined as a locoregional recurrence or distant metastasis. Participants were excluded from data analysis when they had missing data on recurrence status (n = 463) or when they had no surgery (n = 8)

^b^Dietary intake was adjusted for energy using the energy residual method. To improve interpretability, the predicted nutrient intake at the median total energy intake was added to individual residuals [15]

^c^The adjusted model was adjusted for age, sex, stage of disease and pre-diagnostic energy intake

^d^Low-fat dairy included buttermilk and low-fat, reduced-fat or skimmed versions of milk, yoghurt, custard, and soft curd cheese

^e^High-fat dairy included whole and condensed milk, full-fat versions of yoghurt, custard and soft curd cheese, all other cheeses, ice cream, whipped cream, and butter

### Post-diagnostic dairy intake in relation to recurrence and all-cause mortality

As shown in Table 3 and Supplementary Fig. 1, post-diagnostic total dairy intake was not associated with recurrence or all-cause mortality. A higher intake of low-fat dairy after diagnosis tended to be associated with a lower risk of all-cause mortality (HR_Q4 vs Q1_: 0.76, 95% CI 0.53–1.08; P_RCS_: 0.011; Table 3 and Fig. 2B). RCS revealed a significant non-linear association between post-diagnostic low-fat dairy intake and all-cause mortality, with intakes higher than the median of the first quartile (i.e. 20 g/day) associated with a reduced risk of mortality (Fig. 2B). Post-diagnostic low- and high-fat dairy intakes were not associated with recurrence. A higher intake of high-fat dairy after diagnosis was associated with an increased risk of all-cause mortality (HR_Q4 vs Q1_: 1.60, 95% CI 1.10–2.33; P_RCS_: 0.006). Post-diagnostic milk, yoghurt, and cheese intake were not associated with recurrence or all-cause mortality.

**Table 3:** Associations of post-diagnostic energy-adjusted intake of total dairy, low-fat dairy, high-fat dairy, and dairy products with recurrence (n = 1215) and all-cause mortality (n = 1605) in sex-specific quartiles of people with stage I-III colorectal cancer

Dietary variable	Recurrence^a^				All-cause mortality			
	Median energy-adjusted intake [IQR]^b^	n	No. of recurrences/person-years	Adjusted model, HR (95% CI)^c^	Median energy-adjusted intake [IQR]^b^	n	No. of deaths/ person-years	Adjusted model, HR (95% CI)^c^
Total dairy								
Q1	125.3 [92.7, 157.9]	305	31/889	1.00	125.7 [90.8, 157.9]	396	51/2211	1.00
Q2	227.7 [206.4, 257.8]	304	41/899	1.38 (0.86–2.21)	228.1 [206.6, 254.1]	403	59/2186	1.06 (0.73–1.55)
Q3	341.6 [318.2, 365.7]	303	39/842	1.31 (0.81–2.10)	335.1 [303.5, 362.6]	400	60/2161	1.07 (0.73–1.55)
Q4	485.9 [440.2, 606.7]	303	39/853	1.27 (0.79–2.04)	481.4 [434.8, 593.7]	406	65/2272	1.00 (0.69–1.45)
P_trend_	NA	NA	NA	0.47	NA	NA	NA	0.94
Continuous (per 100 g/day)	285.1 [184.7, 396.6]	1215	150/3484	1.06 (0.97–1.16)	282.7 [185.9, 393.7]	1605	235/8829	1.03 (0.95–1.10)
Low-fat dairy^d^								
Q1	23.0 [0.3, 46.7]	302	37/871	1.00	20.1 [− 1.7, 46.2]	396	66/2151	1.00
Q2	120.1 [94.4, 140.1]	305	36/897	0.95 (0.60–1.50)	119.3 [94.9, 139.8]	402	58/2220	0.83 (0.58–1.18)
Q3	221.7 [187.0, 259.4]	304	36/852	1.03 (0.65–1.63)	216.9 [180.6, 255.5]	402	52/2211	0.70 (0.49–1.01)
Q4	382.6 [322.3, 470.0]	304	41/864	1.14 (0.73–1.78)	380.3 [322.6, 469.4]	405	59/2247	0.76 (0.53–1.08)
P_trend_	NA	NA	NA	0.48	NA	NA	NA	0.12
Continuous (per 100 g/day)	161.0 [71.3, 288.0]	1215	150/3484	1.03 (0.94–1.13)	161.2 [72.0, 286.6]	1605	235/8829	*
High-fat dairy^e^								
Q1	29.2 [13.2, 40.9]	307	40/853	1.00	29.2 [13.5, 40.0]	400	43/2213	1.00
Q2	64.7 [54.8, 73.0]	304	33/902	0.80 (0.50–1.30)	64.9 [54.0, 73.0]	403	61/2232	1.27 (0.85–1.89)
Q3	102.9 [90.7, 115.9]	305	32/895	0.77 (0.48–1.24)	102.2 [90.4, 116.2]	405	49/2249	0.94 (0.62–1.44)
Q4	209.7 [168.5, 291.9]	299	45/834	1.02 (0.66–1.57)	202.7 [166.1, 273.6]	397	82/2135	1.60 (1.10–2.33)
P_trend_	NA	NA	NA	0.62	NA	NA	NA	0.010
Continuous (per 100 g/day)	81.2 [48.4, 136.6]	1215	150/3484	1.08 (0.93–1.24)	81.2 [48.5, 135.9]	1605	235/8829	1.18 (1.06–1.32)
Total milk								
Q1	− 0.4 [− 14.4, 8.4]	307	32/863	1.00	− 1.1 [− 15.3, 7.0]	394	57/2106	1.00
Q2	32.9 [25.6, 42.8]	301	42/873	1.31 (0.82–2.10)	31.9 [24.1, 40.5]	401	54/2252	0.79 (0.53–1.17)
Q3	104.6 [78.9, 133.8]	305	30/897	1.01 (0.61–1.67)	101.2 [75.7, 128.6]	403	50/2209	0.78 (0.53–1.15)
Q4	256.2 [191.9, 306.5]	302	46/851	1.39 (0.88–2.19)	237.1 [183.6, 299.5]	407	74/2261	0.99 (0.70–1.42)
P_trend_	NA	NA	NA	0.28	NA	NA	NA	0.54
Continuous (per 100 g/day)	58.8 [16.9, 159.8]	1215	150/3484	1.05 (0.93–1.18)	56.9 [16.4, 155.6]	1605	235/8829	1.02 (0.92–1.12)
Total yoghurt								
Q1	4.5 [− 4.3, 11.7]	301	30/888	1.00	4.2 [-4.4, 12.1]	397	62/2229	1.00
Q2	45.7 [29.4, 58.9]	303	44/862	1.38 (0.86–2.20)	47.4 [29.1, 61.2]	402	62/2171	0.99 (0.70–1.41)
Q3	104.1 [84.9, 121.7]	305	33/897	1.03 (0.63–1.69)	103.8 [82.9, 121.1]	402	56/2215	0.84 (0.58–1.20)
Q4	187.3 [144.2, 266.7]	306	43/836	1.51 (0.96–2.39)	187.8 [143.8, 269.6]	404	55/2214	0.89 (0.62–1.28)
P_trend_	NA	NA	NA	0.39	NA	NA	NA	0.17
Continuous (per 100 g/day)	76.3 [20.5, 136.8]	1215	150/3484	1.07 (0.91–1.27)	75.5 [21.7, 137.2]	1605	235/8829	0.96 (0.83–1.11)
Total cheese								
Q1	8.9 [3.3, 12.9]	303	37/848	1.00	8.1 [2.1, 12.6]	397	55/2133	1.00
Q2	21.2 [18.9, 23.8]	301	47/825	1.37 (0.88–2.12)	21.1 [18.6, 23.4]	403	66/2193	1.02 (0.71–1.48)
Q3	32.9 [29.6, 36.9]	305	34/889	0.92 (0.57–1.46)	32.7 [29.3, 36.5]	403	55/2247	0.86 (0.59–1.25)
Q4	53.1 [46.8, 67.5]	306	32/921	0.83 (0.51–1.33)	52.4 [46.5, 66.4]	402	59/2257	0.89 (0.61–1.29)
P_trend_	NA	NA	NA	0.18	NA	NA	NA	0.40
Continuous (per 30 g/day)	26.3 [16.3, 40.9]	1215	150/3484	0.85 (0.68–1.07)	26.1 [15.9, 40.4]	1605	235/8829	0.93 (0.78–1.11)

*CI* confidence interval, *HR* hazard ratio; *IQR* inter-quartile range. Cut-off points for female quartiles were as follows: 208.8, 317.5 and 418.1 g/day for total dairy; 91.2, 181.8 and 303.1 g/day for low-fat dairy; 61.0, 89.8 and 145.5 g/day for high-fat dairy; 21.7, 52.0 and 153.5 g/day for milk; 45.1, 102.9 and 152.5 g/day for yoghurt; 18.5, 28.2 and 42.4 g/day for cheese. Cut-off points for male quartiles were as follows: 166.9, 263.2 and 375.3 g/day for total dairy; 61.6, 147.3 and 270.6 g/day for low-fat dairy; 42.5, 74.0 and 129.1 g/day for high-fat dairy; 12.7, 59.4 and 155.5 g/day for milk; 14.0, 58.7 and 121.9 g/day for yoghurt; 14.1, 24.4 and 39.4 g/day for cheese

^*^This association was observed to be non-linear in restricted cubic splines. See Fig. 2 for the continuous analysis

^a^Recurrence is defined as a locoregional recurrence or distant metastasis. Participants were excluded from data analysis when they had missing data on recurrence status (n = 419), when they had no surgery (n = 8) or when they had a recurrence before filling out the post-diagnostic FFQ (n = 9)

^b^Dietary intake was adjusted for energy using the energy residual method. To improve interpretability, the predicted nutrient intake at the median total energy intake was added to individual residuals [15]

^c^The adjusted model was adjusted for age, sex, stage of disease and post-diagnostic energy intake

^d^Low-fat dairy included buttermilk and low-fat, reduced-fat or skimmed versions of milk, yoghurt, custard, and soft curd cheese

^e^High-fat dairy included whole and condensed milk, full-fat versions of yoghurt, custard and soft curd cheese, all other cheeses, ice cream, whipped cream, and butter

Except for pre- and post-diagnostic low-fat dairy in relation to all-cause mortality, we observed no evidence of non-linearity in our RCS analyses, and findings of these analyses were in line with the Cox proportional hazard regression analyses (Fig. 2 and Supplementary Figure 1).

## Discussion

This study demonstrated that a higher low-fat dairy intake was associated with a reduced risk of all-cause mortality in people with stage I-III CRC, whereas a higher intake of high-fat dairy was associated with an increased risk of all-cause mortality, both before and after diagnosis. Furthermore, a higher pre-diagnostic intake of yoghurt was associated with a reduced risk of all-cause mortality. Also, to our knowledge, this is the first study to demonstrate that higher pre-diagnostic intakes of total and low-fat dairy were associated with a reduced risk of recurrence.

Our findings on post-diagnostic low- and high-fat dairy intake in relation to all-cause mortality are in line with the results of a recent pooled analysis of the Nurses’ Health Study and Health Professionals Follow-up Study in people with stage I-IV CRC, that also demonstrated a reduced risk of all-cause mortality with higher post-diagnostic low-fat dairy intakes, and an increased risk with higher post-diagnostic high-fat dairy intakes [7]. Previous large cohort studies observed no statistically significant associations between pre- [5, 6] or post-diagnostic [6, 7] total dairy intake and all-cause mortality. Therefore, we stress the importance of considering low- and high-fat dairy separately when studying health effects of dairy.

The mechanisms linking dairy consumption to prognostic outcomes in CRC are not clear and current hypotheses are largely derived from research focussing on CRC aetiology [3, 4]. Calcium has been suggested to reduce CRC risk via binding secondary bile acids and free fatty acids in the colonic lumen, thereby reducing oxidative stress and reactive proliferation of the epithelium [19, 20]. Also, clinical trials have demonstrated that calcium can alter molecular pathways that lead to direct inhibition of cell proliferation, promotion of cell differentiation, and induction of apoptosis in tumour cells [20–25]. Other dairy components, such as conjugated linoleic acid, lactic acid-producing bacteria in fermented dairy, lactoferrin, and vitamin D in fortified dairy foods, may also reduce CRC risk [2, 3, 21, 26, 27]. Previous work from our group showed that, although no clear associations were observed, the HRs for recurrence and all-cause mortality tended to be lower for those with a higher dietary calcium intake [28]. The present study includes a larger study population and more events for recurrence and all-cause mortality. To provide insight into whether observed associations in the current study are independent of dietary calcium intake, we carried out additional exploratory analyses where we adjusted our models for dietary calcium intake (data not shown). Except for the association between cheese intake and risk of recurrence, which changed to a more positive, but still non-significant association, the observed associations did not substantially change. Furthermore, considering the current uncertainty about the biological mechanism linking dairy to CRC outcomes, this study focuses on food groups rather than micronutrients, which is closer to people’s perception of the diet and easier to translate into dietary guidelines.

Our study suggests that pre-diagnostic, but not post-diagnostic, intake of low-fat dairy is associated with a reduced risk of recurrence. A potential explanation for this time-dependent finding could be that pre-diagnostic consumption may well reflect habitual dietary consumption in the decades before cancer diagnosis, implying low-fat dairy may directly or indirectly influence carcinogenesis early in the CRC continuum [9]. Also, methodological issues, such as differences in number of events and duration of follow-up time, or differences in dairy intake could explain this time-dependent finding. For post-diagnostic intake, our study showed only associations with all-cause mortality, not with recurrence, which may suggest that post-diagnostic dairy consumption influences CRC prognosis via other ways than recurrence, such as risk of cardiovascular disease [29].

In our study, a higher pre-diagnostic intake of yoghurt intake was associated with a reduced risk of all-cause mortality. Previous large prospective cohort studies showed no statistically significant association between pre-diagnostic yoghurt consumption and CRC-specific mortality [5, 9]. Possibly, yoghurt consumption influences cardiovascular risk factors rather than CRC [30], explaining why yoghurt may be associated with all-cause mortality in the current study, but not with CRC-specific mortality in other studies. Therefore, future studies should include cause-specific death information when studying associations between yoghurt consumption and CRC prognosis.

We observed no associations between pre- or post-diagnostic total milk or cheese intake and CRC prognosis. Previous studies on milk intake demonstrated a marginal positive [5] or no association [6, 7] between pre-diagnostic total milk intake and mortality. For post-diagnostic intake, the two US cohorts observed a reduced risk of all-cause mortality with higher intakes of low-fat milk [7] and total milk [6]. In contrast to the milk studied in the US cohorts [6, 7], Dutch milk is not fortified with vitamin D. As higher concentrations of 25-hydroxyvitamin D in the blood have previously been associated with improved survival in CRC [31], this might explain the discrepancy between findings. Also, compared to an estimated median milk intake of ~ 100–150 g/day in previous studies, our study population had a relatively low milk intake with a median intake of around 50 g/day. However, our study population contained many non-consumers of milk, and mean intakes were in line with the most recent national food consumption survey [32]. Only two previous cohorts studied cheese separately, and also demonstrated no association between pre- [5] or post-diagnostic [7] cheese intake and mortality in CRC. Future studies should investigate unfortified low-fat milk intake in relation to mortality in CRC.

This study has several strengths, including the prospective study design and the availability of detailed information on cancer treatment, recurrence and both pre- and post-diagnostic dietary intake and supplement use data. Furthermore, considering the current global transition towards more plant-based diets [33–35], it is highly relevant to study potential health effects of dairy in cancer survivorship. A limitation of the current study is the lack of information on the specific causes of death. However, we believe all-cause mortality is a highly relevant study outcome with strong clinical relevance besides the risk of recurrence, as people with CRC are also more prone to develop other chronic diseases compared to the general population [29]. A second potential limitation is the possibility of underestimation of self-reported dietary intake data inherent to the chosen dietary assessment method. However, FFQs are commonly used in cohort studies for feasibility reasons, and literature has previously shown to capture intake of dairy reasonably well by FFQs in general [36–38]. Also, even though we tested for confounding of many different lifestyle factors, we cannot exclude the possibility of residual confounding. Lastly, our participants were primarily Caucasian, limiting the generalizability of our study. Future studies should make an effort to recruit a diverse range of ethnicities.

## Conclusion

This study found that higher pre- and post-diagnostic intakes of low-fat dairy were associated with a reduced risk of all-cause mortality in people with stage I-III CRC, whereas higher intakes of high-fat dairy were associated with an increased risk of all-cause mortality. We also demonstrated that a higher pre-diagnostic intake of low-fat dairy was associated with a reduced risk of recurrence in persons diagnosed with stage I-III CRC. Future research should differentiate between low-and high-fat dairy when investigating associations with recurrence and mortality. When replicated in other large prospective cohorts and ultimately intervention studies, our results can contribute to specific dietary guidelines for CRC survivors who aim to improve their prognosis via lifestyle change.

## Supplementary Information

Below is the link to the electronic supplementary material.

Supplementary file 1: (PDF 66 KB)

## Data Availability

Because the data consist of identifying cohort information, some access restrictions apply, and therefore they cannot be made publicly available. Data will be shared with permission from the acting committee of the COLON study. Requests for data can be sent to dr. Fränzel van Duijnhoven, Division of Human Nutrition and Health, Wageningen University & Research, Netherlands (e-mail: franzel.vanduijnhoven@wur.nl).
