# Supplementary material for: Pre- and post-diagnostic dairy intake in relation to recurrence and all-cause mortality in people with stage I-III colorectal cancer
Source: Eur J Nutr. 2023 Jul 2;62(7):2891–904. doi: 10.1007/s00394-023-03201-0 (PMC10468734; doi:10.1007/s00394-023-03201-0)
Supplement: Supplementary file 1 — (PDF 66 KB) [file 394_2023_3201_MOESM1_ESM.pdf]

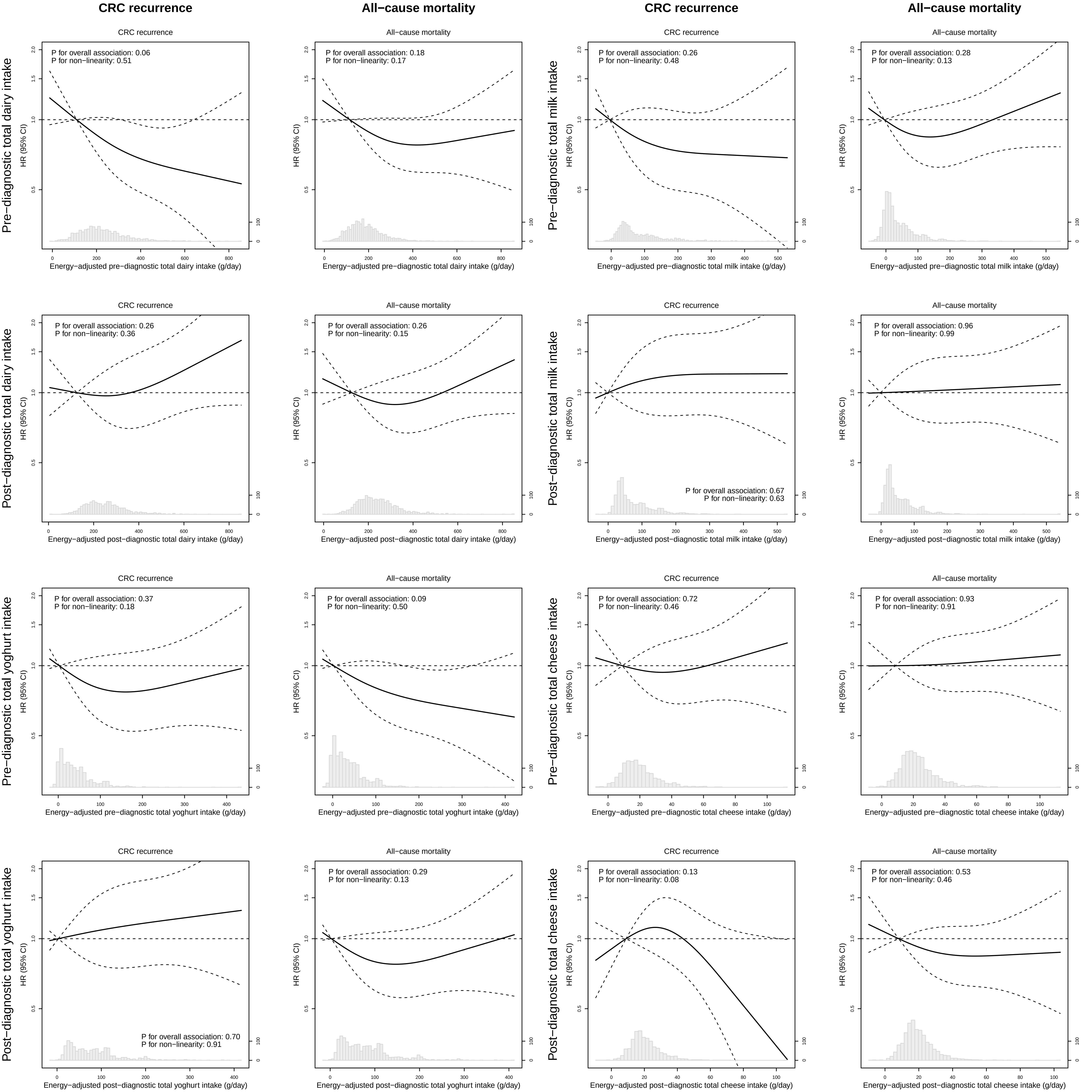

CRC recurrence
All−cause mortality
CRC recurrence
All−cause mortality
Pre−diagnostic total dairy intake
Post−diagnostic total dairy intake
Pre−diagnostic total milk intake
Post−diagnostic total milk intake
Pre−diagnostic total yoghurt intake
Post−diagnostic total yoghurt intake
Pre−diagnostic total cheese intake
Post−diagnostic total cheese intake
HR (95% CI)
P for overall association: 0.06
P for non−linearity: 0.51
Energy−adjusted pre−diagnostic total dairy intake (g/day)
P for overall association: 0.18
P for non−linearity: 0.17
Energy−adjusted pre−diagnostic total dairy intake (g/day)
P for overall association: 0.26
P for non−linearity: 0.48
Energy−adjusted pre−diagnostic total milk intake (g/day)
P for overall association: 0.28
P for non−linearity: 0.13
Energy−adjusted pre−diagnostic total milk intake (g/day)
P for overall association: 0.26
P for non−linearity: 0.36
Energy−adjusted post−diagnostic total dairy intake (g/day)
P for overall association: 0.26
P for non−linearity: 0.15
Energy−adjusted post−diagnostic total dairy intake (g/day)
P for overall association: 0.67
P for non−linearity: 0.63
Energy−adjusted post−diagnostic total milk intake (g/day)
P for overall association: 0.96
P for non−linearity: 0.99
Energy−adjusted post−diagnostic total milk intake (g/day)
P for overall association: 0.37
P for non−linearity: 0.18
Energy−adjusted pre−diagnostic total yoghurt intake (g/day)
P for overall association: 0.09
P for non−linearity: 0.50
Energy−adjusted pre−diagnostic total yoghurt intake (g/day)
P for overall association: 0.72
P for non−linearity: 0.46
Energy−adjusted pre−diagnostic total cheese intake (g/day)
P for overall association: 0.93
P for non−linearity: 0.91
Energy−adjusted pre−diagnostic total cheese intake (g/day)
P for overall association: 0.70
P for non−linearity: 0.91
Energy−adjusted post−diagnostic total yoghurt intake (g/day)
P for overall association: 0.29
P for non−linearity: 0.13
Energy−adjusted post−diagnostic total yoghurt intake (g/day)
P for overall association: 0.13
P for non−linearity: 0.08
Energy−adjusted post−diagnostic total cheese intake (g/day)
P for overall association: 0.53
P for non−linearity: 0.46
Energy−adjusted post−diagnostic total cheese intake (g/day)
